# COVID-19 disease severity in language minorities in Finland: an observational population-based register study

**DOI:** 10.1186/s12889-025-23160-x

**Published:** 2025-05-29

**Authors:** Juulia Pynnönen, Heli Salmi, Mitja Lääperi, Anu Kantele, Ville Holmberg

**Affiliations:** 1 Department of Infectious Diseases, University of Helsinki and Helsinki University Hospital, Helsinki, Finland; 2 Department of Anaesthesia and Intensive Care, University of Helsinki and Helsinki University Hospital, Helsinki, Finland

**Keywords:** COVID-19, Ethnicity, Language, Migrant, Minority, SARS-CoV-2, Laboratory parameter

## Abstract

**Background:**

Migrants and ethnic minority populations appear to have experienced a disproportionate burden of the coronavirus disease 2019 (COVID-19) pandemic. In Finland, a foreign first language is often considered an indicator of foreign ethnicity. We investigated whether hospitalized patients with a foreign first language presented with a more severe COVID-19 upon admission to specialized health care, and if they had a similar in-hospital course of disease compared with those with a domestic first language.

**Methods:**

We conducted a retrospective observational population-based register study, including all patients with confirmed COVID-19 infection admitted to specialized health care and intensive care units (ICUs) in the capital province of Finland between 27 February and 3 August 2020. We examined first values, peak values, and changes in laboratory parameter elevation during hospitalization among different language groups. Studied biomarkers included creatinine, alanine aminotransferase (ALAT), fibrin D-dimer, leukocyte count, thrombocytes, C-reactive protein (CRP), and lactate dehydrogenase (LDH).

**Results:**

First language was registered for 614 (94.9%) patients admitted to specialist care, of whom 131 (21.3%) had a foreign first language. Those with a foreign first language were younger (median 49, IQR 36.5–57, versus 62, 50–75.5, years, *p <* 0.001) and exhibited smaller changes in CRP (22.5, IQR 0–104.75 versus 52, IQR 7.25–125, *p =* 0.031). After adjustment for age and sex, none of the differences remained significant. A foreign first language did not impact the risk of ICU admission (20.1% versus 21.4%, *p =* 0.81). Peak leukocyte count, change in leukocyte count, and all CRP variables were the most significant indicators of ICU admission (*p <* 0.001 for all) and behaved similarly across language groups.

**Conclusions:**

CRP and leukocyte count were the most reliable indicators of more severe COVID-19 disease presentation and ICU admission. Although those with a foreign first language were overrepresented in COVID-19 related hospitalizations, we found no significant differences in laboratory findings based on first language, implying that disease severity among hospitalized patients did not differ between language minorities and the rest of the population.

**Supplementary Information:**

The online version contains supplementary material available at 10.1186/s12889-025-23160-x.

## Introduction

Several studies from Europe and North America have revealed higher rates of COVID-19 infection among ethnic minorities compared to majority ethnic populations [1–6]. Similar observations have been made among migrants in many high-income countries [7]. Suggested explanations for the increased infection rates among ethnic minorities include sociodemographic factors such as heightened workplace exposure, large household size, and neighborhood characteristics [1, 2, 8]. However, the findings have been less consistent when examining COVID-19-related ICU admissions and in-hospital mortality among minority populations [6, 9–11]. Numerous studies have described general risk factors for severe disease, including male sex, advanced age, and several chronic diseases [12–16].

In previous research conducted in the Finnish context, foreign language proficiency or country of birth has been employed as surrogate markers for foreign ethnicity [9]. Consistent with previous observations in some Western countries, minority ethnic populations in Finland have exhibited a higher incidence and increased rates of admission to specialized health care due to COVID-19 infection [1, 5, 9, 17]. Holmberg et al. (2021) explored the impact of language background on COVID-19 outcomes in Finland, finding that individuals with a foreign first language had lower testing rates but higher positivity rates. While they observed higher odds of hospitalization among foreign-language speakers, this was largely explained by testing disparities. No significant differences were found in ICU admission, mortality, or clinical outcomes such as SOFA scores, mechanical ventilation use, or length of ICU stay between language groups.

The majority of studies related to laboratory findings in COVID-19 have focused on identifying microbiological markers indicative of severe infection, or prognostic tools. High leukocyte counts, thrombocytopenia, high lactate dehydrogenase (LDH), high C-reactive protein (CRP), high procalcitonin, and high interleukin-6 have been associated with severe COVID-19 disease [15, 16, 18–20]. Males have been found to exhibit higher inflammation parameter elevations than females irrespective of outcome [21]. Limited research on the association between ethnicity and these prognostic laboratory parameters has demonstrated some variation according to ethnicity [22–25]. However, the results have been inconsistent, highlighting the role of environmental factors contributing to these disparities [22, 26].

Our study seeks investigate to extend on earlier work by examining whether laboratory markers of disease severity and progression differ between individuals with foreign and domestic first languages. By using inflammatory biomarkers, we aim to identify any subtle differences in disease progression that might not be apparent in clinical outcomes alone, and to determine if there are external factors present, that influence the disease progression across language groups. This investigation is important for understanding whether language-related disparities persist at the level of disease severity, despite similar clinical outcomes.

## Methods

### Setting

Finland is a Nordic welfare country that had a population of 5 525 292 by the end of 2019 [27]. COVID-19 was classified as a dangerous communicable disease during the study period. As such, all inpatient and ICU treatment for COVID-19 were provided by the public health care system free of charge for all inhabitants, encompassing Finnish residents, migrants, and tourists. In the Helsinki region, all necessary care is always provided for undocumented migrants. The Finnish population register maintains records of residents’ country of birth, nationality, and self-reported language information. In this study, we used self-reported first language as an indicator of immigrant background.

Finland has two official languages: Finnish and Swedish. Additionally, there is a third minority domestic language, Sami, predominantly spoken in the Lapland region. At the end of 2019, 7.5% of the Finnish population reported a first language other than Finnish, Swedish, or Sami. The Uusimaa region in Southern Finland had a population of 1 689 725 inhabitants, of whom 78.2% had Finnish, 7.7% Swedish, and 137 persons (0.00%) Sami as a reported first language. Approximately 14% had a foreign first language, and 439 individuals (0.00%) lacked language data [27]. Additionally, language data might be missing for undocumented migrants and temporary visitors within the study cohort. All residents are assigned personal identification codes, which were used to link health care data for this study.

### Study population

We conducted a retrospective observational population-based registry study that included all individuals with COVID-19 infection confirmed by the Helsinki University Hospital laboratory services and admitted to specialized in-hospital care in the capital province of Finland between 27 February and 3 August 2020. Previously institutionalized patients who were mainly treated in primary care facilities were excluded from the study.

### Retrieval of data

Laboratory and clinical data were extracted from the electronic patient records of the Helsinki University Hospital district. The collected information comprised age, sex, first language, and basic laboratory test results. Data were collected on patients treated exclusively in regular wards and on patients admitted to ICU during their hospitalization. The studied laboratory results included creatinine, alanine aminotransferase (ALAT), fibrin D-dimer, leukocyte count, thrombocytes, C-reactive protein (CRP), and lactate dehydrogenase (LDH). We restricted the inclusion of laboratory test results to the time period spanning 7 days before and 30 days after the laboratory confirmation of COVID-19 disease. We then retained the first recorded test result and the highest recorded test result for each parameter within the defined time frame for each patient. For thrombocytes, we also included the lowest recorded result within the same time frame. Individuals with missing data on language and sex were excluded from subsequent analyses involving these variables.

### Statistical analyses

We described the data using counts with percentages and medians with interquartile ranges (IQRs). Pairwise comparisons between groups were done using Mann–Whitney U tests and Fisher tests. We also used logistic regression models to investigate the differences in biomarkers between patients speaking foreign and domestic native languages, adjusting for age and sex. An interaction term between sex and biomarker was included in the models to account for potential sex-based differences in disease severity. Since patients who spoke a foreign language were generally younger than those who spoke the native language, age adjustment was necessary. Additionally, sex was included in the models as it could influence both the biomarkers and the clinical outcomes. We further investigated the effect of the biomarkers on patients admitted to ICU and those not admitted. We modelled all continuous variables using restricted cubic splines to allow non-linear effects and fit multivariable models adjusting for age, sex and language group. We also included a language group-biomarker interaction term, to allow the models to capture any differential effect of the biomarker depending on language. *P*-values below 0.05 were considered significant. All analyses were done with R version 4.2.2 using rms and ggplot2 packages.

### Ethics approval and consent to participate

The study was a retrospective registry study by design, obviating the need for permission from the Ethical Committee of Helsinki University Hospital. Institutional approval was obtained with no prerequisite of patient consent (HUS/1049/2020/× 4 and HUS/157/2020/× 94), authorizing the patients to be included.

## Results

### Characteristics of the cohort

During the study period, 647 patients with confirmed COVID-19 disease were admitted to specialized hospital care. Data on language were missing for 33 patients (5.1%), and on sex for 2 patients (0.3%). Out of the 614 patients with data on first language, 489 received care in regular wards and 125 were treated in intensive care units (ICUs). The distribution of age, sex, and first language in the study population and between regular wards and ICUs is presented in Table 1. Patients with a foreign first language were younger (median 62, IQR 50–76 versus 49 years, IQR 37–57, *p* < 0.001). The difference in age narrowed in repeated analyses with under 70-year-os, but those with a foreign first language were still younger (median 54, IQR 45–62, versus 47, IQR 36–56 years, *p* < 0.001). First language did not exert a significant risk for the likelihood of ICU admission (20.1% versus 21.4%, *p* = 0.81). Males were more likely to be admitted to ICU than females (23.2% versus 16.8%, *p* = 0.049).

**Table 1:**
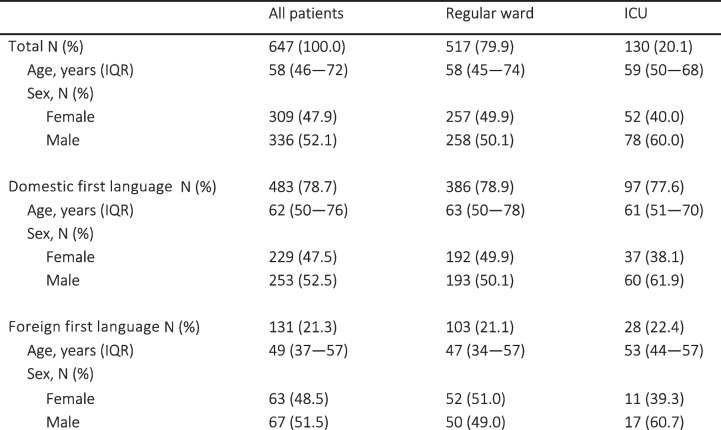
Demographics of patients from language minorities in regular ward and intensive care unit (ICU) cohorts

### Comparison of regular ward and ICU

**Fig. 1:**
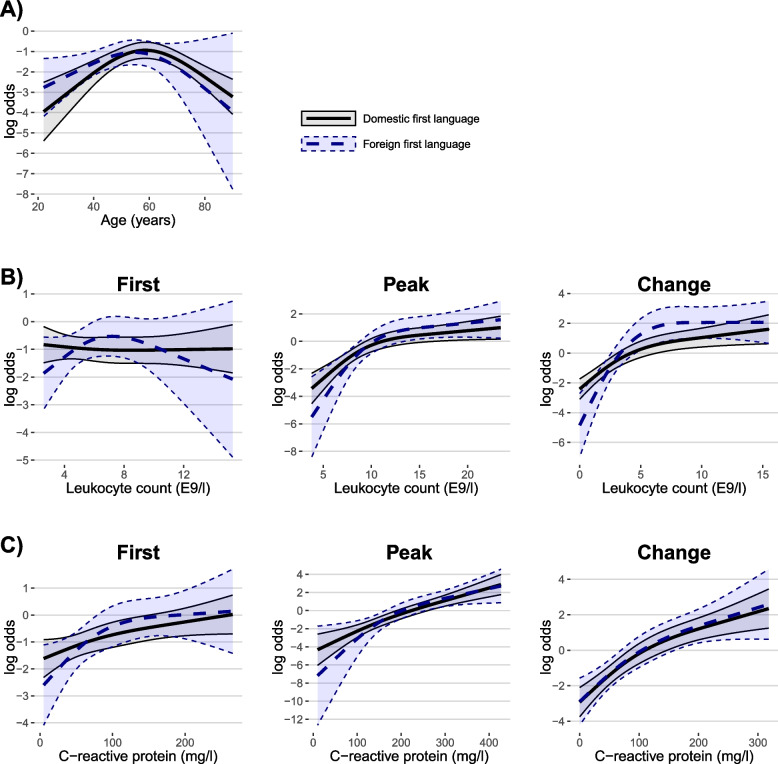
Log odds estimate plots from multivariate models comparing patients treated in ICU versus regular ward. Risk plots of multivariable models for patients requiring intensive care unit treatment instead of regular ward, adjusted for age and sex, including the language group – biomarker interaction

In multivariable regression models investigating the impact of language group, sex and age on the odds for ICU-admission, only age was significant (X2 28.4, df = 1, *p* < 0.001), as demonstrated in the log odds plot found in Additional file 1. Laboratory results with medians for patients treated in regular wards versus ICUs are provided in Table 2. High rates of missing values were observed in LDH (67.2%), D-dimer (39.9%), and ALAT (16.5%). Abnormal values were more prevalent among those treated in ICUs. However, most variables lost significance in multivariable models, adjusted for both age and sex, including language group-biomarker interaction. The difference in multivariable regression analyses remained the most significant for peak CRP, peak leukocyte count and change in leukocyte count (*p* < 0.001 for all). Out of the parameters with no significant data gaps, first CRP value remained the only statistically significant first value after multivariable analyses. The results of multivariable regression analyses for laboratory profiles comparing patients requiring ICU treatment with those receiving regular ward treatment, and the influence of first language on laboratory parameter results are described in Table 3. Log odds plots for multivariable regression models are found in Fig. 1 and Additional file 2, visualizing the non-linear influence of parameter elevation and age on the odds of ending up in ICU. Overall, language group did not have any significant influence on the laboratory profile. The change in leukocyte count was the only parameter with a noticeable interaction with first language, although this effect was only barely significant (*p* = 0.049).

**Table 2:**
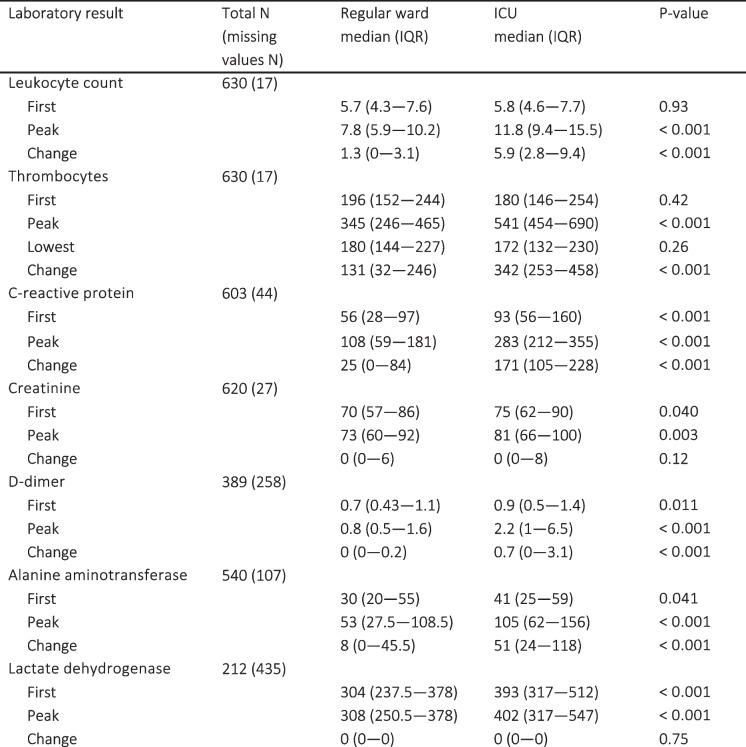
Laboratory results and their prevalence of missing values in regular wards versus intensive care units

*ICU* Intensive care unit

Significant *p*-values are bolded

**Table 3:**
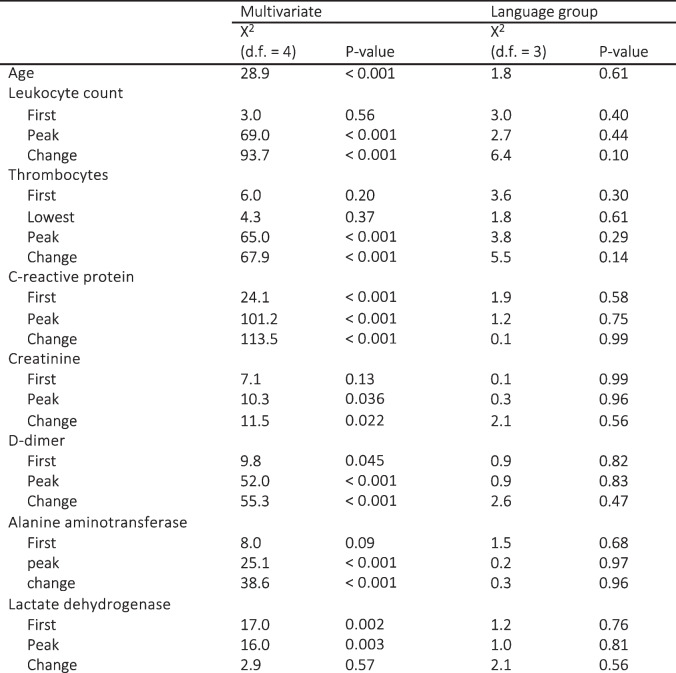
Results of multivariable regression models comparing patients treated at regular wards versus intensive care units

Models were adjusted for age, sex, language group, and language group – biomarker interaction and modelled non—linearly using restricted cubic splines. The results for the difference between language groups are specified in its own paragraph. Wald test was used to test the overall significance of the variable. Significant *p*-values are bolded

### Comparison between language groups

In pairwise comparison, those with a foreign first language had smaller changes in CRP (52, IQR 7.25–125 versus 22.5, IQR 0–104.75, *p* = 0.031), lower first d-dimer (0.8, IQR 0.5–1.3 versus 0.6, IQR 0.4–1.1, *p* = 0.0065) and lower first (73, IQR 60–90, versus 67, IQR 55–83.75, *p* = 0.0080) and peak creatinine values (77, IQR 63–99 versus 71, IQR 58–88.75, *p* = 0.0087). When repeating analyses with under 70-year-olds, thrombocytes remained the only parameter with significantly lower values among foreign language speakers (peak thrombocytes 419, IQR 292.5–542 versus 368.5, IQR 250–503.5, *p* = 0.032; change in thrombocytes 210.5, IQR 68.5–338.5 versus 136, IQR 40.75–289.75, *p* = 0.027), although the differences were barely significant. None of the biomarkers remained significant when adjusted for both age and sex. Results of univariate and multivariable regression analyses of the laboratory results in language groups are shown in Table 4. Log odds plots describing the multivariable models can be found in Additional file 3.

**Table 4:**
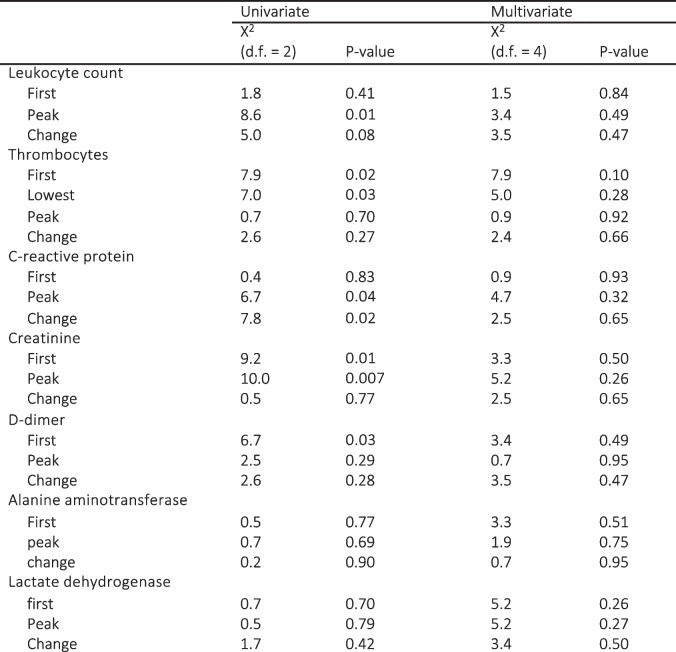
Results of univariate and multivariable logistic regression models comparing language groups

Multivariable models included adjustments for both age and sex and also the interaction between biomarker and sex. Variables were modelled non—linearly using restricted cubic splines. Wald test was used to test the overall significance of the variable. Results suggest that differences in the biomarkers were due to age and sex. Significant *p*-values are bolded

## Discussion

Previous studies have shown that minority populations experience higher incidences of COVID-19 and are more likely to be admitted to specialized healthcare [1, 5, 9, 17]. Thus, it was of paramount interest to ascertain whether the severity of the disease and its outcomes during their hospital stay would differ from those with a domestic first language.

### Laboratory parameters

Our study presents data collected during the first wave of the COVID-19 pandemic, when the vast majority of the population was immunologically naïve to SARS-CoV-2, with no prior infection or vaccination. Immunological profiles have since then changed considerably due to exposures to the virus and vaccination programs [28]. In our study, CRP emerged as the best overall early predictor for ICU admission, with higher first values increasing the odds for ICU admission. Although, similar to previous research, also the first values of LDH and D-dimer proved significant in multivariable models, their respective predictive values in this study were weaker due to the substantial proportion of missing data [15, 19, 20]. Overall, the magnitude of laboratory parameter abnormality correlated positively with ICU admission for all parameters. Among these laboratory results, CRP and leukocyte count exhibited the most significant peak values and changes, and thus proved to be the most reliable late indicators for severe COVID-19 disease, regardless of age. This biomarker profile correlates with earlier studies concerning prognostic laboratory tests for severe COVID-19 infection. Elevated CRP levels at admission, along with a rise during hospitalization, are widely recognized as indicators of severe disease [16, 19, 20]. Similarly, high leukocyte counts and an increase in leukocytes have been associated with severe disease [19, 20].

### Age distribution

Those with a foreign first language were not only overrepresented at hospitals, but also proved younger and had milder laboratory findings.

They presented with less prominent inflammation development and CRP elevation, in addition to better renal function, though this difference was small and clinically likely not that significant. Overall, these findings suggest a trend toward milder disease based on laboratory parameters. Younger individuals have generally fewer comorbidities and better physical fitness than the older patients, who accordingly are at higher risk for severe COVID-19. Interestingly, all disparities in laboratory findings between the language groups faded in multivariable analyses, suggesting that the initial differences in laboratory profiles were attributed to age differences: those in the foreign language group being younger than those with a domestic first language. Similar age differences exist also in the general population in Finland. For instance, in 2019, 76% of those with a foreign first language in Finland were of working age, whereas the corresponding figure for domestic language speakers was 61% [27]. While young age serves as a protective factor against severe COVID-19 disease, young adults have exhibited markedly higher incidence rates, placing younger populations at higher risk for infection. However, the necessity for hospital care became more pronounced after the age of 40 [17].

### Impact on ICU admission

First language appeared not to affect the odds of ICU treatment once hospitalized. Most laboratory parameters behaved similarly in both language groups and log odds plots demonstrated similar trends. No significant difference between language groups was found in multivariable regression models. The change in leukocyte count was the only parameter with an interaction with first language. Consistently, the log odds plot demonstrated a slightly steeper elevation of odds for ICU admission with rising change in leukocyte count for those with a foreign first language. However, the overall difference did not prove significant, and the interaction was only slightly significant, suggesting that this finding may not be relevant. Our data did not unveil differences in the in-hospital course of COVID-19 disease by language group. This concurs with previous studies demonstrating similar odds ratios for ICU admission, disease severity, and ICU outcomes across all language groups, suggesting that ICU admission criteria and treatment modalities were consistent during the study period [9].

### Impact on the course of disease

While it has been established that ethnic and language minorities in many countries have suffered from higher incidences and increased attack rates for COVID-19 infection [1–6], our study demonstrates that minority status does not impact the course of disease once the patient is admitted to quality health care. This aligns with previous studies, indicating similar outcomes between minorities and the general population once in care, suggesting instead that lower testing rates among those with a foreign first language led to only the more severe cases being detected, and consequently, a bigger portion of the tested being hospitalized [9, 22]. In contrast to some international studies, some evidence, albeit not significant, has suggested a lower COVID-19 mortality among ethnic minorities in Finland [14, 17, 29]. This could be explained by the younger age distribution among language minorities in Finland or by the so-called healthy migrant effect, with healthier individuals with fewer comorbidities being presumably more prone to migration, hence, leading to increased survival rates.

### Strengths and limitations

One of our main strengths is the high-quality population-based register data. Language data for most of the study population was attained from the Population Information System in Finland that keeps a comprehensive registry of inhabitants'self-reported first language. Importantly, as the language data is self-reported, the information may better reflect ethnic background than actual language skills, as many second-generation migrants and migrants that have lived in Finland for longer periods are often fluent also in Finnish. For this reason, the results should not be viewed as strictly language-dependent but also as reflecting the ethnicity-related features of the study population. Additionally, some migrants may have recorded Finnish as their first language, thus potentially diminishing slightly the observed differences. This also reduces the impact of any small, ethnicity-related variations in laboratory profiles, such as differences in neutrophil counts. However, potential ethnic variations in laboratory parameters should still be considered a possible limitation [30]. However, we do not consider this a significant limitation, as the relationship between inflammatory responses and ethnicity is complex and multifactorial [26, 31]. Relying on broad ethnic categorizations may, therefore, be overly simplistic and potentially counterproductive in capturing the nuanced effects of ethnicity on disease outcomes, particularly in the heterogenous population in our study. Finally, our choice of non-linear statistical modelling allowed us to explore general patterns and trends between language groups. We perceive this as a significant strength of the study, as it gives a more authentic representation of trends when results are not forced into linear models regardless of their true shape. For this reason, we did not set exact numeric threshold values for laboratory findings. However, we feel that our choice of statistical method was adequate since our aim was to simply determine whether a significant difference exists in the laboratory profiles of our study groups. However, the use of multiple tests increases the risk of Type I errors in our analyses.

## Conclusion

Even though ethnic language minorities had higher infection rates and were overrepresented in specialized hospital care during the first wave, they did not have a more severe course of COVID-19. When accounting for age and sex, the COVID-19 severity and course of disease did not differ between domestic and foreign language groups once admitted to specialized hospital care. This suggests that disease progression was similar across language groups. Consequently, it can be inferred that treatment modalities and evaluation metrics were likely appropriate throughout the study period. The differences observed initially in the laboratory profiles between domestic and foreign language populations were likely due to younger age in the group of language minorities. Our data suggest that the unequal distribution of adverse outcomes in COVID-19 among minority populations is likely primarily attributed to multifactorial pre-hospital factors, not to differences in the severity of the disease in this group.

## Supplementary Information

Supplementary Material 1.

Supplementary Material 2.

Supplementary Material 3.

## Data Availability

The datasets used and/or analyzed during the current study are available from the corresponding author on reasonable request.

## Abbreviations

ICU: Intensive care unit
ALAT: Alanine aminotransferase
LDH: Lactate dehydrogenase
CRP: C-reactive protein
