# Supplementary figures and images for: COVID-19 disease severity in language minorities in Finland: an observational population-based register study
Source: BMC Public Health. 2025 May 29;25:1978. doi: 10.1186/s12889-025-23160-x (PMC12121010; doi:10.1186/s12889-025-23160-x)

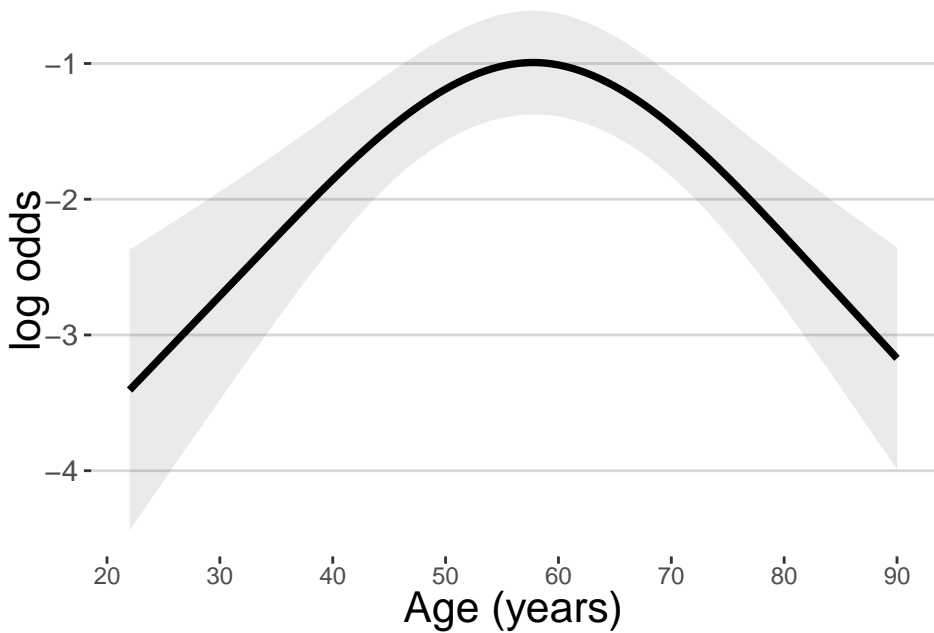

Supplement: Supplementary file 1 — Supplementary Material 1. [file 12889_2025_23160_MOESM1_ESM.pdf]

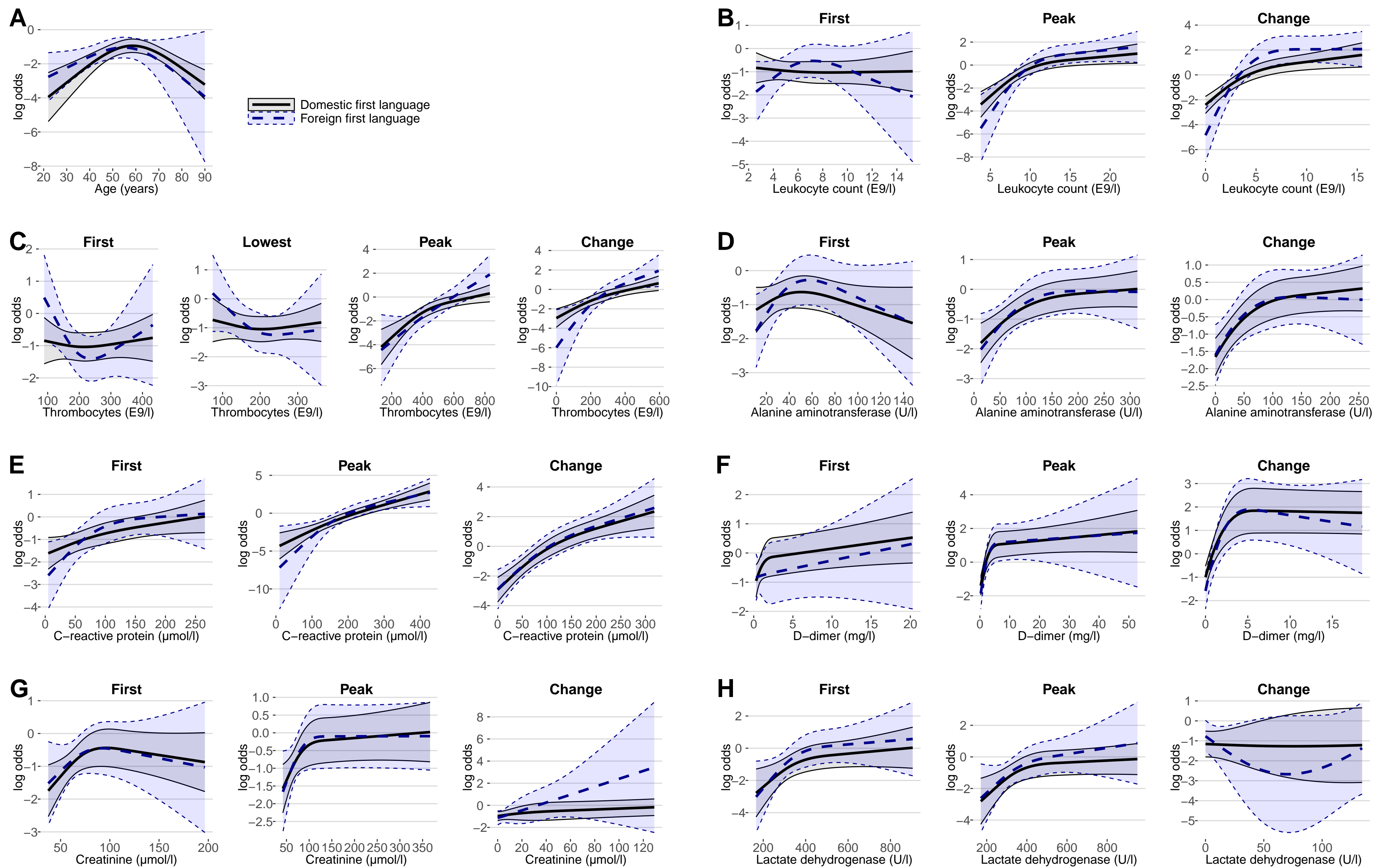

Supplement: Supplementary file 2 — Supplementary Material 2. [file 12889_2025_23160_MOESM2_ESM.pdf]

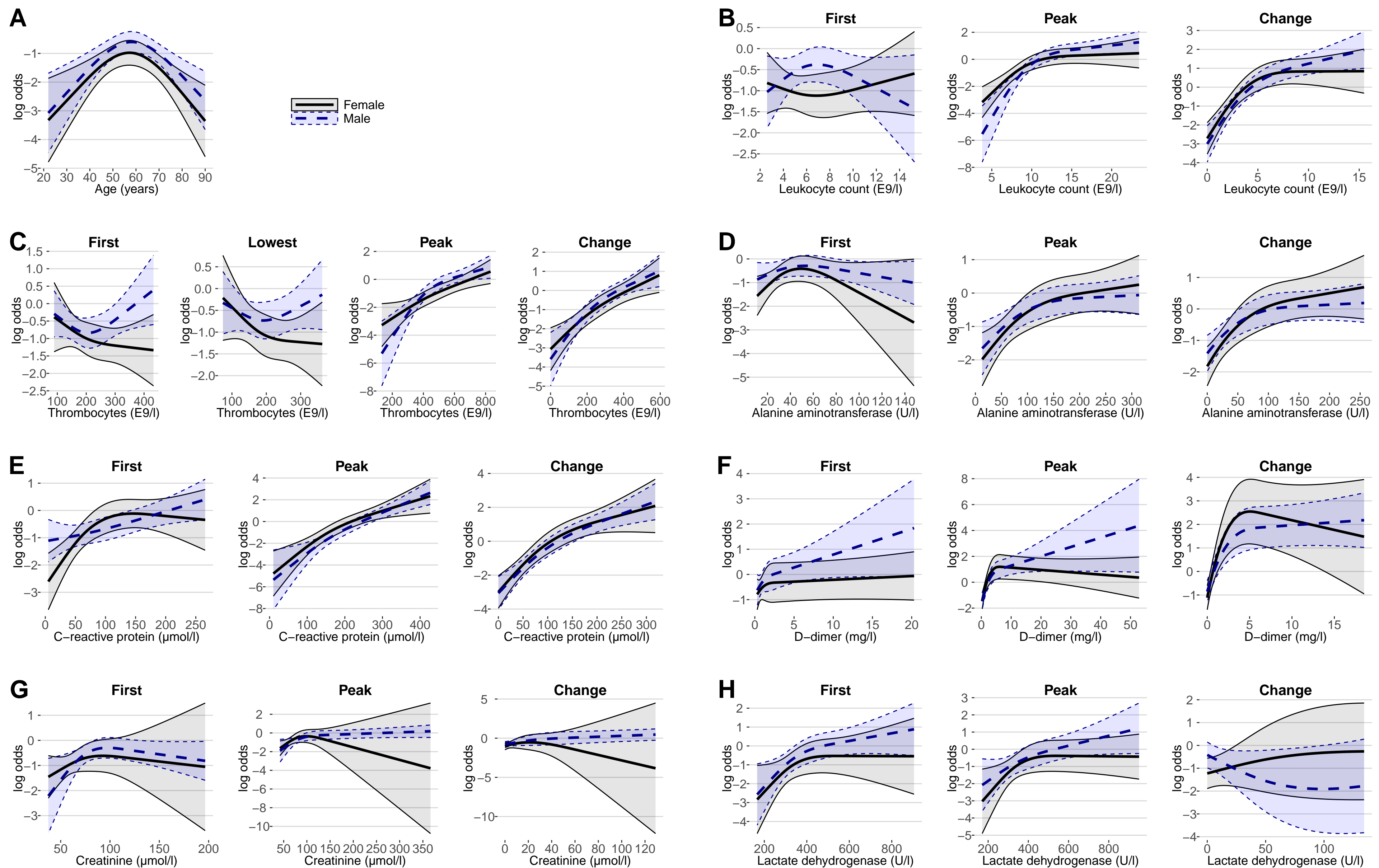

Supplement: Supplementary file 3 — Supplementary Material 3. [file 12889_2025_23160_MOESM3_ESM.pdf]
